# MicroRNA-19b-3p regulates nasopharyngeal carcinoma radiosensitivity by targeting TNFAIP3/NF-κB axis

**DOI:** 10.1186/s13046-016-0465-1

**Published:** 2016-12-05

**Authors:** Teng Huang, Li Yin, Jing Wu, Jia-Jia Gu, Jian-Zhong Wu, Dan Chen, Hong-Liang Yu, Kai Ding, Nan Zhang, Ming-Yu Du, Lu-Xi Qian, Zhi-Wei Lu, Xia He

**Affiliations:** 1 The Fourth Clinical School of Nanjing Medical University, Nanjing, Jiangsu China; 2 Department of Radiation Oncology, Nanjing Medical University Affiliated Cancer Hospital, Cancer Institute of Jiangsu Province, 42 Bai Zi Ting Road, Nanjing, Jiangsu 210000 China; 3 Research Center of Clinical Oncology, Nanjing Medical University Affiliated Cancer Hospital, Cancer Institute of Jiangsu Province, Nanjing, Jiangsu China

**Keywords:** microRNA-19b-3p, Nasopharyngeal carcinoma, Radiosensitivity, Apoptosis

## Abstract

**Background:**

Nasopharyngeal carcinoma (NPC) is among the most common squamous cell carcinoma in South China and Southeast Asia. Radiotherapy is the primary treatment for NPC. However, radioresistance acts as a significant factor that limits the efficacy of radiotherapy for NPC patients. Growing evidence supports that microRNAs (miRNAs) play an important role in radiation response.

**Methods:**

Real-time quantitative PCR was used to analyze the expression of miR-19b-3p in NPC cell lines and NP69. miR-19b-3p expression profiles in NPC tissues were obtained from the Gene Expression Omnibus database. The effect of miR-19b-3p on radiosensitivity was evaluated by cell viability assays, colony formation assays and in vivo experiment. Apoptosis and cell cycle were examined by flow cytometry. Luciferase reporter assay was used to assess the target genes of miR-19b-3p. Expression of target proteins and downstream molecules were analyzed by Western blot.

**Results:**

miR-19b-3p was upregulated in NPC and served as an independent predictor for reduced patient survival. Radioresponse assays showed that miR-19b-3p overexpression resulted in decreased sensitivity to irradiation, whereas miR-19b-3p downregulation resulted in increased sensitivity to irradiation in vitro. Moreover, miR-19b-3p decreased the sensitivity of NPC cells to irradiation in vivo. Luciferase reporter assay confirmed that TNFAIP3 was a direct target gene of miR-19b-3p. Knockdown of TNFAIP3 reduced sensitivity to irradiation, whereas upregulation of TNFAIP3 expression reversed the inhibitory effects of miR-19b-3p on NPC cell radiosensitivity. Mechanistically, we found that miR-19b-3p increased NPC cell radioresistance by activating the TNFAIP3/ NF-κB axis.

**Conclusions:**

miR-19b-3p contributes to the radioresistance of NPC by activating the TNFAIP3/ NF-κB axis. miR-19b-3p is a determinant of NPC radioresponse and may serve as a potential therapeutic target in NPC treatment.

**Electronic supplementary material:**

The online version of this article (doi:10.1186/s13046-016-0465-1) contains supplementary material, which is available to authorized users.

## Background

Nasopharyngeal carcinoma, which arises from the nasopharynx epithelium, is a common head and neck malignancy among the Southeast Asian populations. Radiotherapy is identified as the primary and only curative treatment for nasopharyngeal carcinoma [1]. Although advanced radiation technology is available, the relapse rate of nasopharyngeal carcinoma is as high as 82%. Radioresistance is a major cause of treatment failure, leading to incomplete cure, recurrence, and metastasis [2].

miRNAs are a class of endogenous single-stranded small noncoding RNA molecules that act as negative post-transcriptional regulators of target gene expression [3]. Previous studies indicate that miRNAs directly affect radioresistance by interfering with specific pathways [4], including sensing DNA damage [5], repair of DNA double-strand breaks (DSBs) [6], cell cycle checkpoint activation [7], formation of reactive oxygen species [8] and apoptosis [9], and autophagy [10]. miR-19b-3p belongs to both the miR-17-92 and miR-106-363 clusters, which play significant roles in proliferation and cell survival [11, 12]. In particular, miR-19b-3p has been validated as an oncogene in several human malignancies [12]. However, the mechanism underlying the role of miR-19b-3p in NPC radioresistance remains elusive.

Here, we report that miR-19b-3p was upregulated in NPC tissues and associated with worse patient prognosis. In addition, we confirmed that miR-19b-3p could affect the radiosensitivity of NPC cells by targeting the 3′-UTR of TNFAIP3 and consequently activating the NF-κB pathway, leading to increased NPC radioresistance in vitro. miR-19b-3p possibly acts as a valuable target for subgroups of patients that might benefit from personalized therapeutic strategies.

## Methods

### Patients and samples

miR-19b-3p expression profiles in NPC tissues were obtained from Gene Expression Omnibus (GEO) (www.ncbi.nlm.nih.gov/geo/). Archived patient samples in The Cancer Genome Atlas (TCGA) database (https:// tcga-data.nci.nih.gov and https:// genome-cancer.ucsc.edu) were selected. Exclusion criteria were as follows: 1) patients without radiation therapy; and 2) samples without completed data for analysis. Receiver operating characteristic (ROC) curve analysis was performed to determine cutoff score for differentiating between low and high miR-19b-3p levels among patients [13].

### Cell lines and culture conditions

Five NPC cells (CNE1, CNE2, 5-8F, 6-10B and HNE1) and NP69 were originated from Research Center of Clinical Oncology of the Affiliated Jiangsu Cancer Hospital, Nanjing Medical University, Nanjing, China. The cells were propagated in RPMI 1640 medium (Hyclone, Logan, UT, USA) containing 10% FBS (Gibco BRL, Gaithersburg, MD, USA) and 1% antibiotic (Gibco BRL), and incubated at 37 °C with CO2 saturated.

### Irradiation

Irradiation was delivered at room temperature using a 6 MeV electron beam generated by a linear accelerator at a dose rate of 400 cGy/min. A compensation glue with 1.0 cm thickness covered the cell culture containers. The source-to-skin distance was 100 cm [14].

### RNA sample preparation

Total RNA was extracted using TRIzol reagent (Invitrogen, Burlington, ON, Canada) according to manufacturer’s protocol. RNA yield and purity were determined by measuring the absorbance (Abs) at 260 and 280 nm. Only RNA samples with Abs260nm/Abs280nm ratio >1.8 were used.

### Cell transfection

CNE-1 and CNE-2 cells were transfected with miR-19b-3p mimic/inhibitor/TNFAIP3-siRNA (RiboBio Guangzhou, China) according to manufacturer’s protocol. To determine the efficiency of miRNA mimic/inhibitor/TNFAIP3-siRNA, the expression of miR-19b-3p/TNFAIP3 was assessed with a qRT-PCR detection system (Bio-Rad, Hercules, CA, USA) and Western blot apparatus.

### Quantitative reverse transcription–polymerase chain reaction analysis

Total RNA was purified using TRIzol reagent (Invitrogen, Carlsbad, CA) according to manufacturer’s instructions. Then, 2 μg of total RNA was reverse-transcribed using Moloney murine leukemia virus reverse transcriptase (Promega, Madison, WI, USA) according to manufacturer’s instructions. Expression levels of mature miRNAs were amplified using SYBR Green quantitative RT-PCR (qRT-PCR) on an ABI7300 real-time PCR machine (Applied Bio-systems). The expression of TNFAIP3 and β-actin was examined using the following specific primers: 5′-TGTGTATCGGTGCATGGTTTTA-3′ and 5′-TCCTCAGGCTTTGTATTTGAGC-3′ and 5′-GGACTTCGAGCAAGAGATGG-3′ and 5′-AGCACTGTGTTGGCGTACAG-3′, respectively. All reactions were performed in triplicate for each sample. Fold changes for miR-19b-3p and TNFAIP3 expression levels were calculated using the 2^-ΔΔCt -^method.

### Cell viability assay

Cell viability was actualized according to literature [15]. Cell counting kit-8 (Beyotime, China) was used according to the manufacturer’s instructions to determine the growth curves for both cell lines. In brief, cells were cultured at 1.5 × 10^3^ cells per well in triplicate in 96-well plates. Following a 12 h culture, the cells were exposed to various doses of radiation depending on the aim of the experiment. Absorbance values were expressed as percentages relative to the controls.

### Colony-forming assay

Clonogenic survival assays were actualized according to literature [16]. Briefly, cells were plated in 60 mm^2^ culture dishes at various cell densities (3 × 10^2^–2 × 10^3^ cells/well) for 12 h and were exposed to ionizing radiation (IR) in doses of 0, 2, 4, 6 and 8 Gy. The cells were cultured for an additional 8–12 days, and colonies were stained with Giemsa. Surviving colonies (a colony was defined as >50 cells) were counted. Radiation dose–response curves were created by fitting the data to the linear quadratic equation *S* = *e* ^-*α*D-*β*D^2^. Area under the curve (AUC) represents the mean inactivation dose (MID). The radiation protection factor (RPF) was calculated by dividing the MID of the test cells by the MID of control cells.

### In vivo experiment

Six week old female nude mice were obtained from Medical Center of Yangzhou University. (Yangzhou, China). The nude mice were subcutaneously injected into the left inguinal with 5 × 10^6^ CNE1 cells respectively. When the xenograft volumes reached 80 mm^3^, all mice were divided into 4 groups (*n* = 6 mice each), including miR-19b-3p agomir group (CNE1/miR-19b-3p agomir group), control agomir group (CNE1/ control agomir group), combination group 1 (CNE1/ miR-19b-3p agomir + RT group), combination group 2 (CNE1/ control agomir + RT group). 5 nmol miR-19b-3p agomir or control agomir (RiboBio) in 50 μl saline buffer was intratumorally injected into the tumor mass at multiple sites per mouse, and next day a 5 Gy dose of ionizing radiation was delivered to the combination group 1 and combination group 2. After 14 days, the combination group 1 and combination group 2 received another 5 Gy dose of ionizing radiation. 5 nmol miR-19b-3p agomir or control agomir (RiboBio) was injected two times a week. All groups followed for about 4 weeks. Tumor volume (in mm^3^) was examined by caliper measurements every 3 to 4 days and calculated by using the modified ellipse formula (volume = length × width^2^/2). Nude mice were sacrificed through decapitation as well as xenograft tumors were rapidly removed after death.

### Target prediction for miRNA candidates

Identification of the predicted target mRNA genes of miRNA provides the basis for understanding miRNA functions. Thus, candidate target genes of miR-19b-3p were analyzed using the miRNA target prediction program PicTar (http://pictar.mdc-berlin.de/) and miRNA.org (http://www.microrna.org/microrna/home.do). Prediction values were calculated to estimate the binding affinities of the miRNAs and their predictive target genes.

### Western blot analyses

After 48 h of transfection, cells were extracted and prepared in modified RIPA buffer (Beyotime, Shanghai, China). Total protein was extracted, and protein concentration was quantified using a BCA protein assay kit (Beyotime, Shanghai, China). A total of 20 mg of protein from each sample was used for Western blot analysis. The primary antibodies used in this study included monoclonal anti-TNFAIP3 (1:1000; Abcam, HK), anti-NF-κB/p- NF-κB (1:1000; Cell Signaling Technology, USA), anti-Bcl-2 (1:1000; Cell Signaling Technology, USA), anti-Bax (1:1000; Cell Signaling Technology, USA), and anti-capase-3/c-capase-3 (1:1000; Cell Signaling Technology, USA). β-actin was used as the loading control. Immunoreactive bands were visualized using ECL detection reagent (Millipore, Billerica, MA, USA). All data analyses were repeated thrice independently.

### Luciferase reporter construction and luciferase assays

A segment of the human TNFAIP3 3′UTR region containing the predicted binding site for miR-19b-3p based on the TargetScan database (http://www.targetscan.org) was synthesized and inserted into the pCDNA3.1 luciferase reporter vector. The sequence of pri-miR-19b-3p also was inserted into the pCDNA3.1 plasmid. To generate the TNFAIP3 3′UTR mutated reporter, a number of nucleotides in the TNFAIP3 3′UTR that were complementary to the seed region nucleotides of miR-19b-3p were mutated. Cells of 293 T line were plated at a density of 1.5 × 10^6^ cells in 24-well plates 24 h before transfection. Reporter plasmids and miR-19b-3p expression plasmids were transfected using the Lipofectamine 2000 reagent (Invitrogen) according to manufacturer’s protocol. The cells were lysed 48 h after transfection and their luciferase activity was assayed using the Picagene Dual SeaPansy luminescence kit (Toyo Ink, Tokyo, Japan) according to standard protocols [12]. All experiments were performed independently thrice. Luciferase activity was normalized to total protein concentration.

### Flow cytometric analysis of the cell apoptosis in response to irradiation

After 36 h of transfection, CNE1 and CNE2 cells were seeded in 6-well plates (5–7 × 10^5^ cells/well and each cell line had a parallel hole) for 12 h and three parallel holes were exposed to 6 Gy IR. Cells were harvested after culture for 3 days and washed twice with ice-cold PBS. Annexin V–FITC/PI staining was used to detect apoptotic cells. Three individual experiments were repeated.

### Pathway analysis

The candidate target genes of miRNAs were analyzed using the miRNA target prediction program RNAhybrid (http://bibiserv.techfak.uni-bielefeld.de/rnahybrid/). KEGG pathway analyses were performed to identify significant signal transduction pathways or enriched metabolic pathways in target gene candidates compared with the whole reference gene background.

### Statistical analysis

Student’s *t*-test and one-way ANOVA statistical analyses were performed with GraphPad Prism 5.0 software and SPSS 13.0. Data were expressed as mean ± standard deviation from at least three separate experiments. Differences were considered significant at *P* < 0.05.

## Results

### miR-19b-3p is upregulated in NPC and associated with worse patient prognosis

**Fig. 1:**
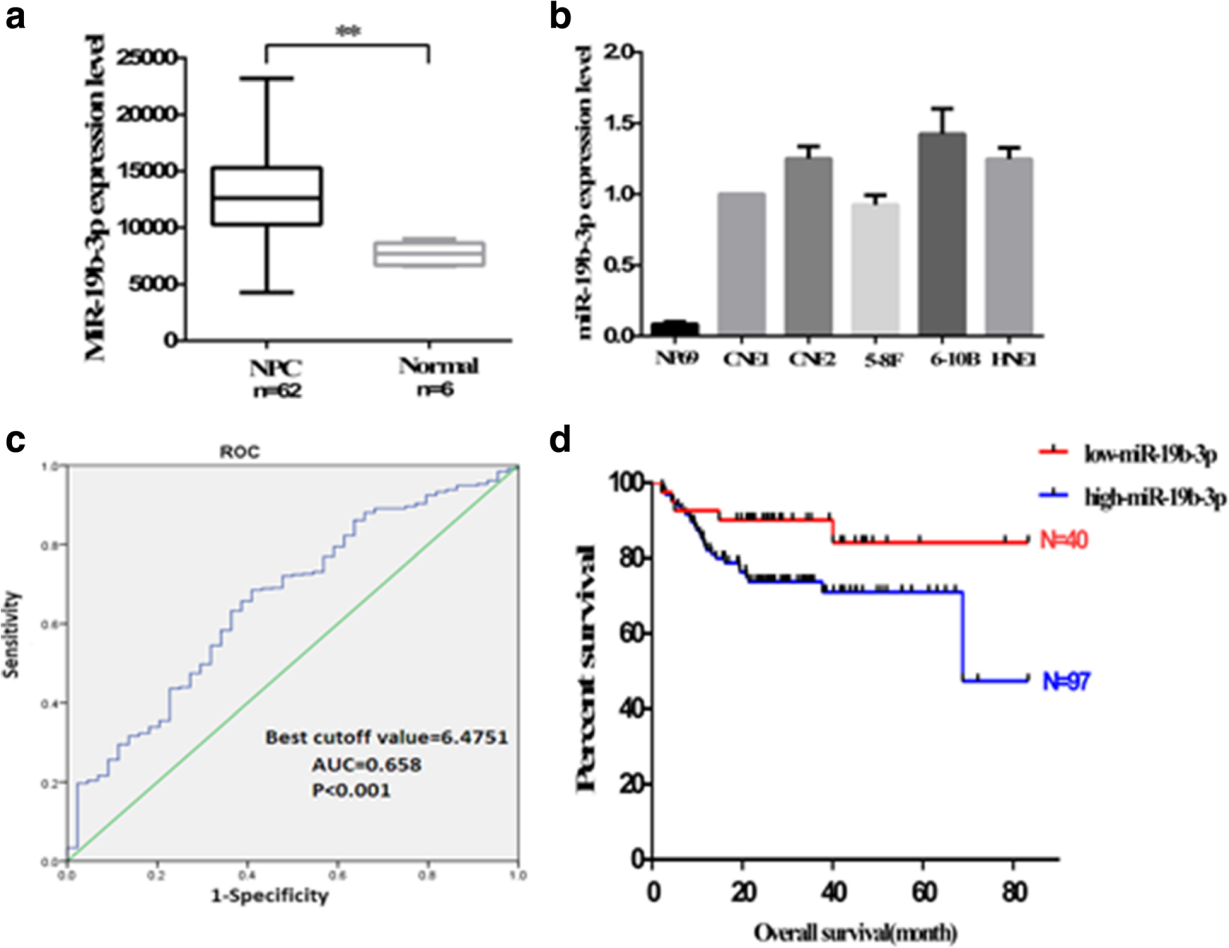
Expression levels of miR-19b-3p in NPC and correlation of miR-19b-3p expression levels with patient survival. **a** Expression levels of miR-19b-3p in normal and NPC tissues. **b** Expression of miR-19b-3p was upregulated in NPC cells. **c** ROC analysis was performed to determine the cutoff value of miR-19b-3p that could differentiate between the NPC patients with low and high miR-19b-3p levels (*P* < 0.001; area under the ROC curve, 0.658; cutoff value, 6.4751). **d** Based on the expression levels of miR-19b-3p Kaplan–Meier survival analysis, patients with high miR-19b-3p expression had significantly worse overall survival than those with low miR-19b-3p expression. The log-rank test was used to calculate *p* value

miR-19b-3p expression profile was obtained using the GEO database for human nasopharyngeal carcinoma samples. Specifically, nasopharyngeal carcinoma (*n* = 62) expressed significantly more miR-19b-3p than normal nasopharyngeal samples (*n* = 6) (GSE36682) (Fig. 1a). The mRNA level of miR-19b-3p in five NPC cell lines (CNE1, CNE2, 5-8F, 6-10B, and HNE1) and NP69 were investigated by qRT-PCR (Fig. 1b). NP69 expressed lower levels of miR-19b-3p than the five NPC cell lines. Radiotherapy is identified as the primary treatment for NPC, but not much information is available on miR-19b-3p’s function in radioresistance. To further investigate the role of miR-19b-3p in radioresistance, we analyzed data retrieved directly from TCGA. The cutoff value of miR-19b-3p was determined by receiver-operating characteristic analysis, which was used to differentiate between low and high miR-19b-3p levels among patients (Fig. 1c). Kaplan–Meier survival analysis of patients was performed based on the expression levels of miR-19b-3p and revealed that miR-19b-3p level negatively correlated with overall survival of patients (Fig. 1d). Univariate Cox regression analysis confirmed that miR-19b-3p expression level and lymph node classification significantly affected overall survival of patients. Multivariate Cox regression analysis showed that high miR-19b-3p expression was an independent predictor for the reduced overall survival of patients (Additional files 1 and 2) (Table 1). Taken together, these data indicate that miR-19b-3p possible play a significant role in NPC radioresistance.

**Table 1:** Univariate and multivariate analyses of prognostic factors for overall survival using Cox proportional hazards regression model (*N* =137)

Variable	Overall survival			
	Univariate analysis		Multivariate analysis	
	HR	95% CI	HR	95% CI
Gender				
Male	1.507	0.617–3.68	1.289	0.515–3.226
Female	1		1	
Age (y)				
≤61	0.898	0.444–1.819	0.847	0.414–1.732
>61	1		1	
Primary tumor (T) stage				
T_1_-T_2_	0.785	0.368–1.674	0.759	0.351–1.641
T_3_-T_4_	1		1	
Lymph node (N) classificaction				
N_0–1_	0.418^*^	0.205–0.851	0.532	0.252–1.124
N_2–3_	1		1	
Neoplasm histologic grade				
G_1_-G_2_	0.739	0.348–1.572	0.838	0.382–1.837
G_3_-G_4_	1		1	
Mir-19b-3p expression level				
Low	0.3^*^	0.104–0.863	0.337^*^	0.114–0.992
High	1		1	

*p < 0.05

### Ectopic expression of miR-19b-3p decreases sensitivity to irradiation

**Fig. 2:**
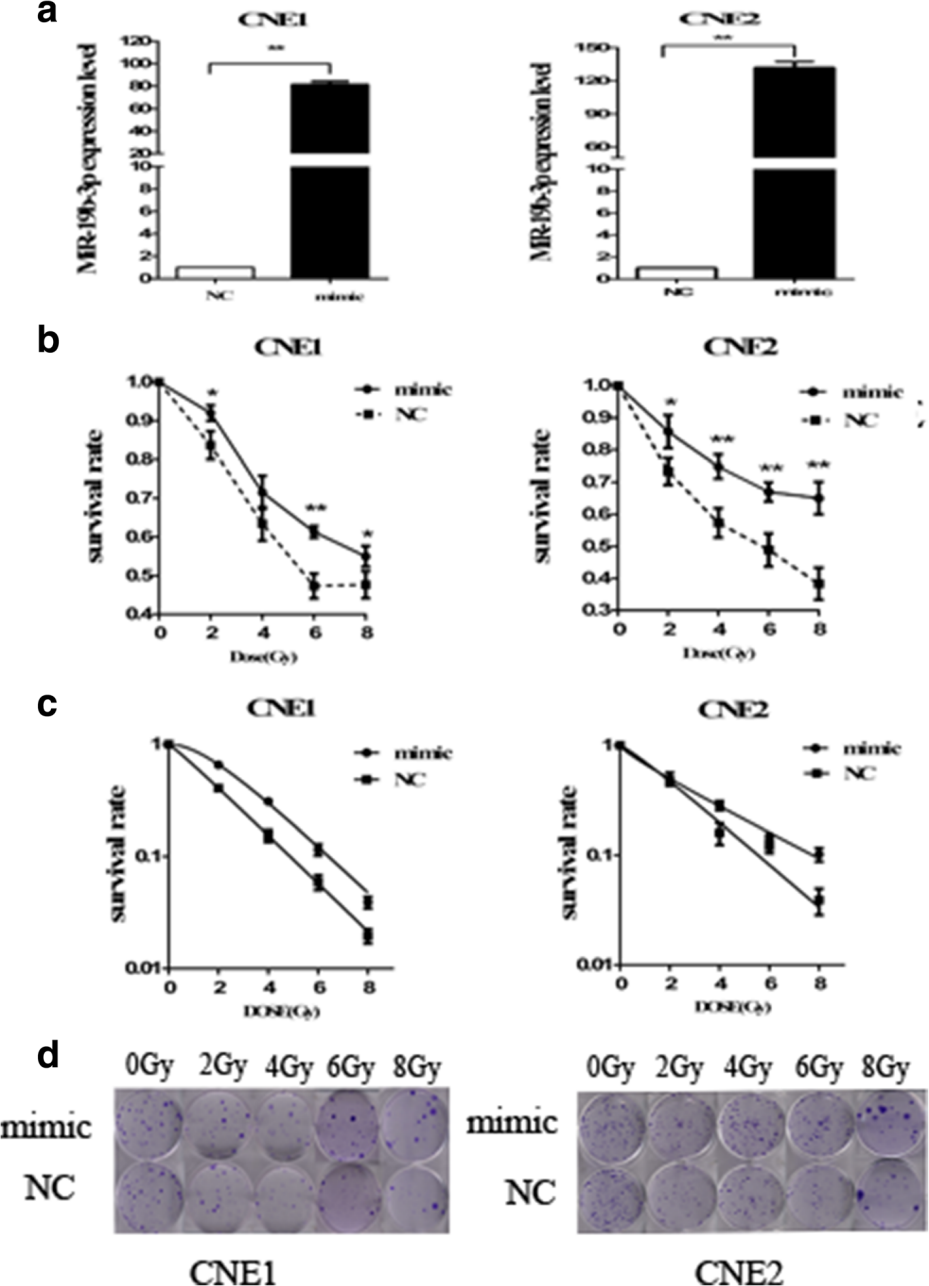
miR-19b-3p increased radioresistance of NPC cells. **a** miR-19b-3p upregulated expression in transfected cells. **b** Survival rates of different cell groups were examined using CCK-8 assays in CNE-1 and CNE-2 cells after 2,4,6,8 Gy irradiation. **c** A clonogenic survival assay shows that transfection of miR-19b-3p mimic increased NPC cells radioresistance compared with control group after 2,4,6,8 Gy irradiation. **d** A representative image of colony formation in CNE-1 and CNE-2 cells exposed to different doses of IR

To investigate the potential mechanism behind the role of miR-19b-3p in NPC radioresistance, we overexpressed miR-19b-3p in CNE-1 and CNE2 cells by transfected with miR-19b-3p mimic. QRT-PCR assays revealed that miR-19b-3p was efficiently upregulated 81.6 ± 2.5 times in CNE-1 cells and 132 ± 5.29 times in CNE2 (Fig. 2a). CCK-8 assay demonstrated increased survival rates of CNE-1 and CNE2 cells with miR-19b-3p overexpression (Fig. 2b). Moreover, clone survival assay showed that upregulation of miR-19b-3p expression markedly decreased radiosensitivity in the NPC cells [AUC 3.20 (CNE1 mimic) vs. 2.26 (CNE1.NC), RPF = 1.42 AUC 2.86 (CNE2.mimic) vs. 2.51 (CNE2.NC), RPF = 1.14; *p < 0.05*] (Fig. 2c, d). The overexpression of miR-19b-3p could significantly decrease the sensitivity of NPC cells.

### Inhibition of miR-19b-3p increases sensitivity to irradiation

**Fig. 3:**
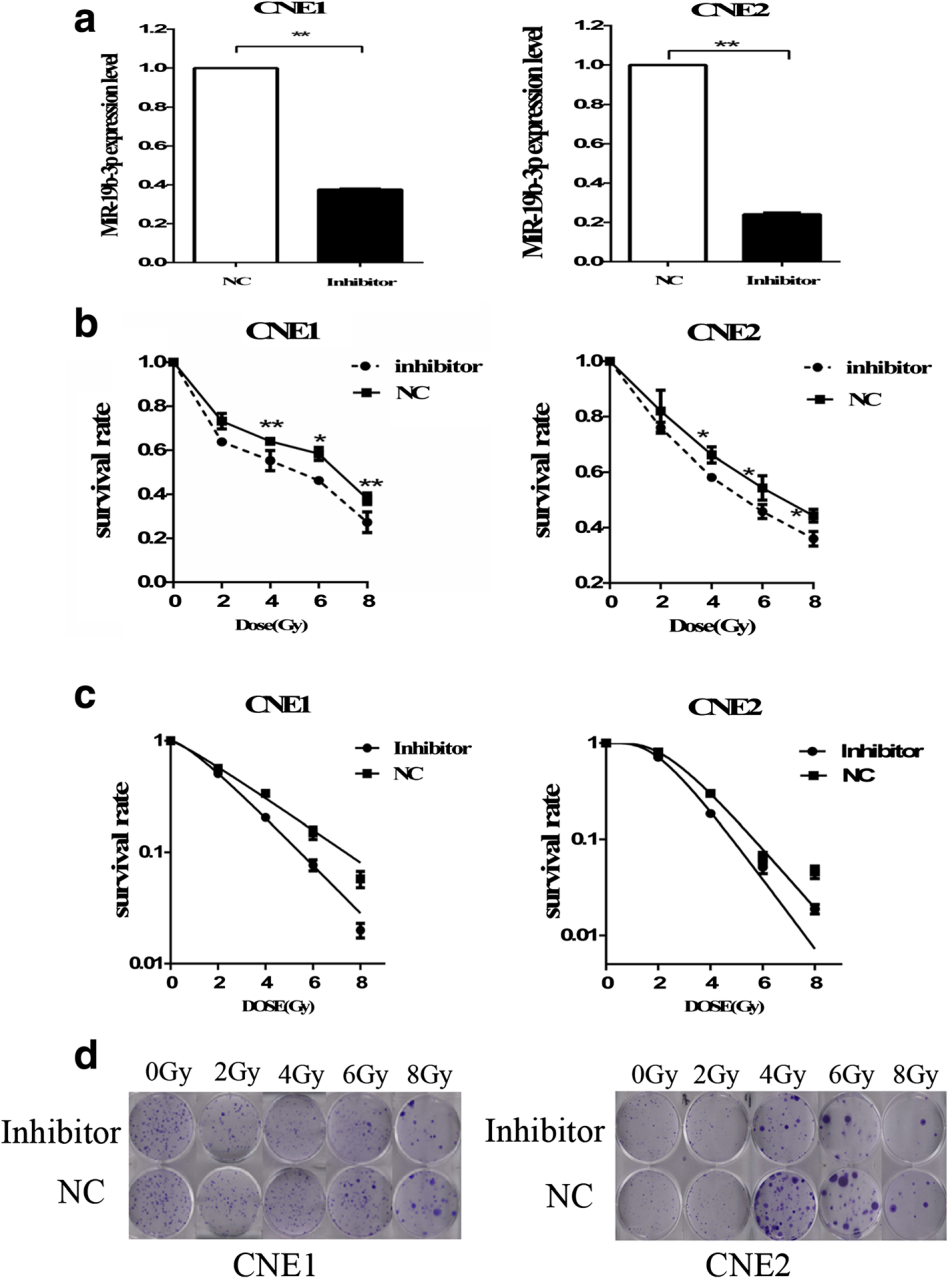
Inhibition of miR-19b-3p increased radiosensitivity of NPC cells. **a** Downregulated miR-19b-3p expression in transfected cells. **b** Survival rates of different cell groups after irradiation were examined using CCK-8 assays after 2,4,6,8 Gy irradiation. **c** Clonogenic survival assay shows that transfection of miR-19b-3p inhibitor increased NPC cell radiosensitivity compared with control groupafter 2,4,6,8 Gy irradiation. **d** Representative image of colony formation in CNE-1 and CNE-2 cells exposed to different doses of IR

Following miR-19b-3p overexpression in CNE-1 and CNE2 cells, we then suppressed miR-19b-3p expression in CNE1 and CNE2 cells. qRT-PCR confirmed that miR-19b-3p expression was decreased to 62 ± 0.6% in CNE1/anti-miR-19b-3p cells and 76 ± 0.85% in CNE2/ anti-miR-19b-3p (Fig. 3a). CCK-8 assay revealed that the CNE1 and CNE2 cells with lower miR-19b-3p level had reduced survival capacity, following irradiation stimulation (Fig. 3b). Furthermore, clone survival assay showed that downregulation of miR-19b-3p expression increased radiosensitivity in the NPC cells [AUC 2.60 (CNE1.inhibitor) vs. 3.16 (CNE1.NC), RPF = 0.82 AUC 2.92 (CNE2.inhibitor) vs. 3.32 (CNE2.NC), RPF = 0.88; *p < 0.05*] (Fig. 3c and d). Taken together, these data confirm that knockdown of miR-19b-3p could decrease the resistance of NPC cells to irradiation.

### miR-19b-3p decreased the sensitivity of NPC cells to irradiation in vivo

**Fig. 4:**
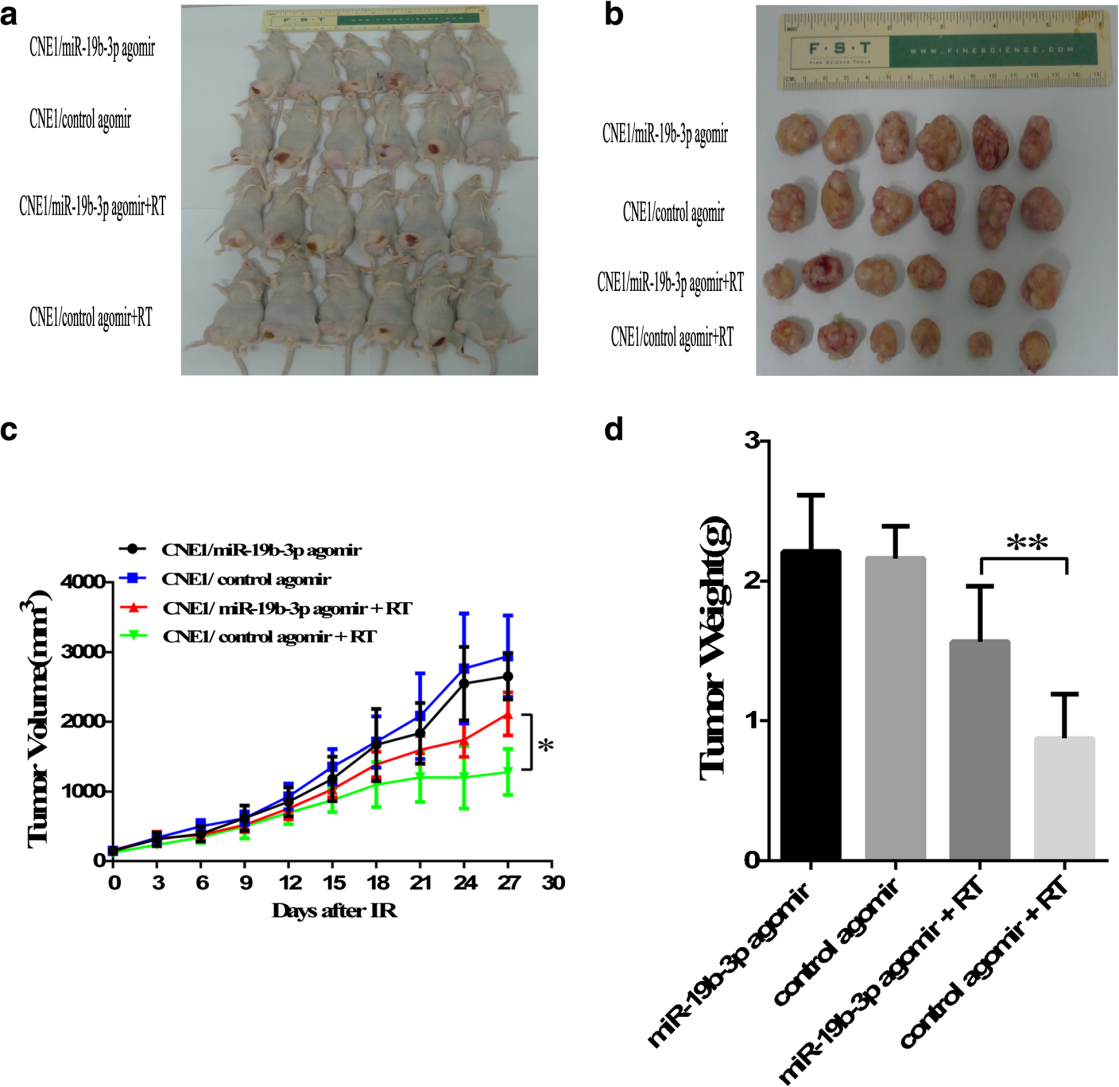
Overexpression of miR-19b-3p influenced the radiosensitivity of CNE1 cells in vivo. **a** and **b**. Representative images of xenograft tumors in different groups of nude mice with or without irradiation, before (left) or after (right) excision. **c** Xenograft tumor growth in different groups after IR. **d** Quantitative determination of xenograft tumor weight of the excised tumors in different groups at the end of the research. *n* = 6 for each groups.*p < 0.05,**p < 0.01

The in vitro results revealed that overexpression of miR-19b-3p can influence radiosensitivity in NPC cells, we furtherly investigated whether miR-19b-3p has a similar effect in vivo. Nude mice were injected with CNE1 cells. We found that miR-19b-3p did not affect tumor growth and weight of group without IR (*p* = 0.31, *p* = 0.79), but could significantly increase tumor growth and weight compared to that in miR-control group after exposure to irradiation (*p* = 0.0011, *p* = 0.0073) (Fig. 4). These results are consistent with our in vitro results.

### miR-19b-3p depressed apoptosis after irradiation stimulation

**Fig. 5:**
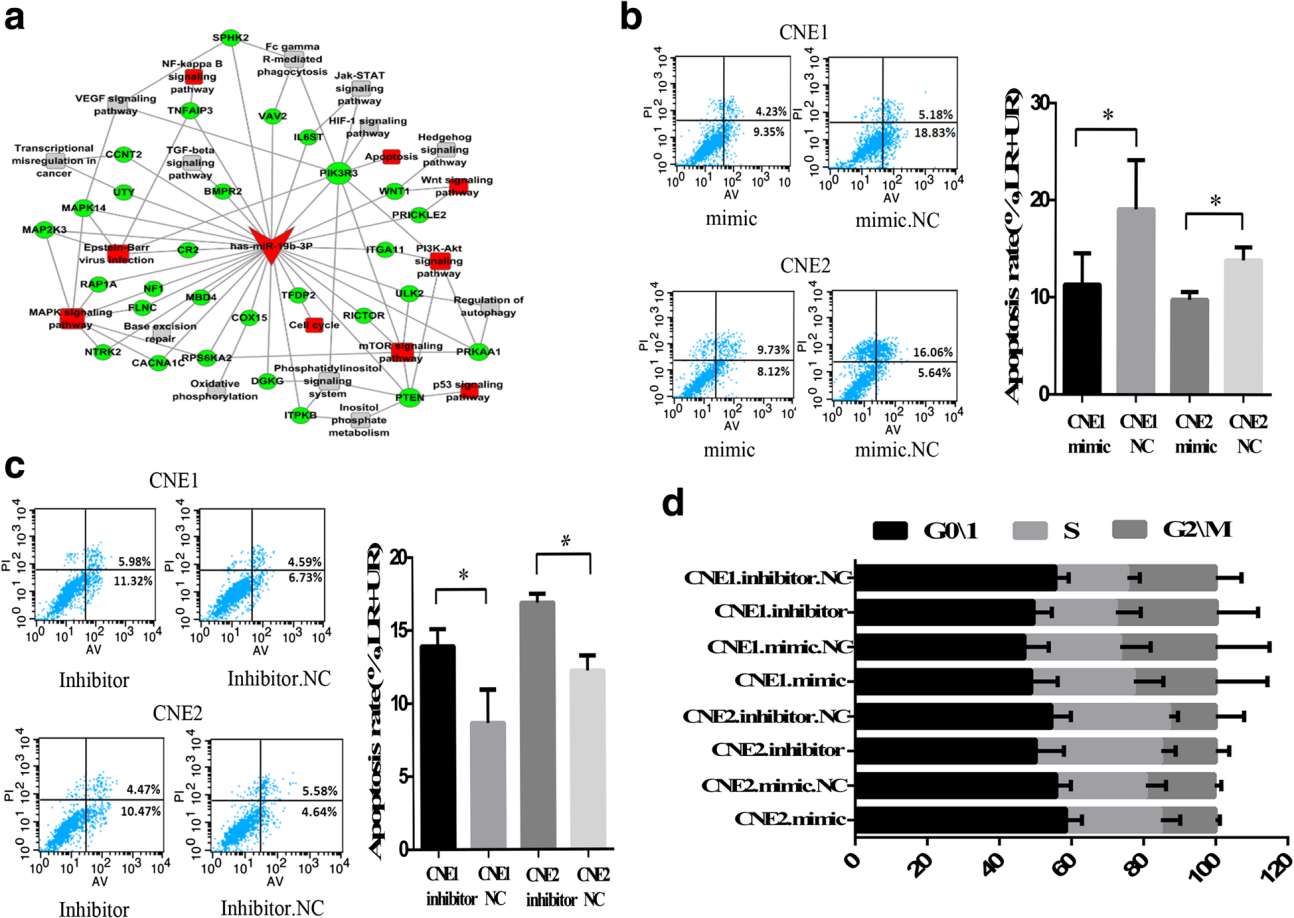
miR-19b-3p decreased apoptosis rate after irradiation stimulation. **a** miRNA-target gene pathway regulatory network was constructed. **b** Apoptotic changes in CNE-1 and CNE-2 cells transfected with mimic after exposure to 4 Gy IR for 72 h. Quantification of the percentage of apoptotic cells (right) is shown. **c** Apoptotic changes in CNE-1 and CNE-2 cells transfected with inhibitor after exposure to 4 Gy IR for 72 h. Quantification of the percentage of apoptotic cells (right) is shown. **d** Alterations in the cell cycle in transfected NPC cells subjected to 4 Gy IR after 72 h. Data are expressed as mean ± SD of three independent experiments

To investigate the potential role of miR-19b-3p in NPC radiobiology, pathway analysis of miR-19b-3p was performed (Fig. 5a). Results showed that miR-19b-3p may play a role in several radioresistance-associated pathways, such as NF-κB, Wnt, and P53 signaling pathways, which are closely related to apoptosis and cell cycle. Thus, both apoptotic and cell cycle effects of miR-19b-3p were assessed by flow cytometry. We found that miR-19b-3p overexpression significantly decreased the percentage of apoptosis in CNE1 and CNE2 cells after irradiation relative to controls. Conversely, CNE1 and CNE2 cells with reduced miR-19b-3p significantly increased the percentage of apoptosis (Fig. 5b and c). However, miR-19b-3p had no effect on the cell cycle of the NPC cells after irradiation stimulation. The correlation between cell cycle and radioresistance in miR-19b-3p may be diminished because of cell arrest after irradiation [17] (Fig. 5d).

### miR-19b-3p regulates TNFAIP3 expression by targeting the 3′-UTR of TNFAIP3

**Fig. 6:**
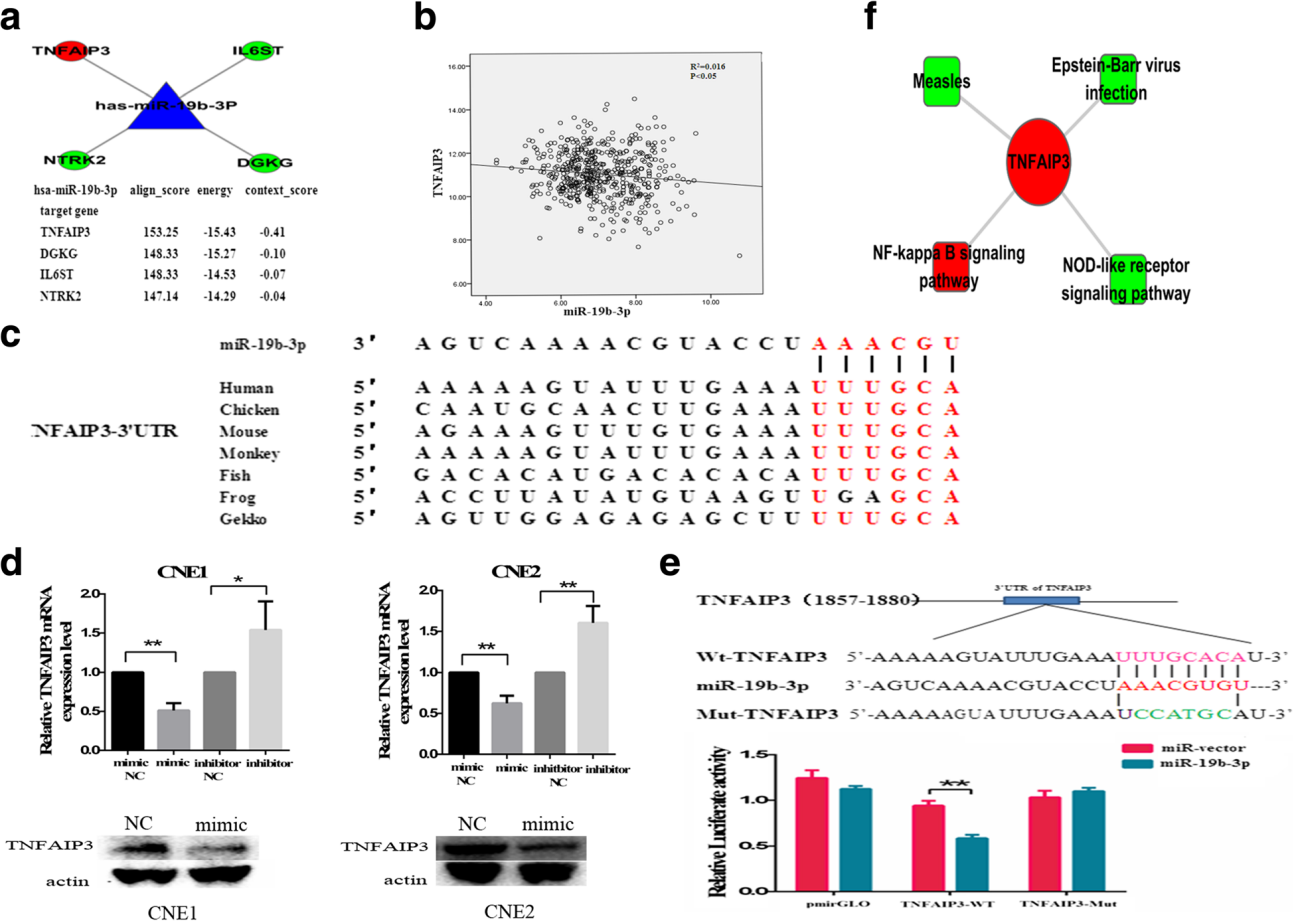
miR-19b-3p regulated TNFAIP3 expression by targeting the 3′-UTR of TNFAIP3. **a** Four candidate target genes of miR-19b-3p were predicted through a miRNA target prediction website. Align score, energy, and context score are all indicated. **b** A negative correlation was found between TNFAIP3 and miR-19b-3p in patient tissues. **c** Sequence alignment of the miR-19b-3p nucleotide sites with the 3′UTR of TNFAIP3 mRNA of different species. **d** TNFAIP3 expression and miR-19b-3p overexpression in transfected cells decreased TNFAIP3 protein expression. **e** The 293 T cells were transfected with miR-19b-3p or the miR-control vector and were co-transfected with the control luciferase reporter plasmid pmirGLO or with this reporter plasmid containing the TNFAIP3 wt 3′-UTR or the TNFAIP3 mut 3′-UTR. Luciferase activity was assayed 48 h later. **f** Biological functions of TNFAIP3 were subjected to KEGG pathway analyses

Identification of the predicted target genes of miRNA provides the basis for understanding miRNA functions. Candidate miR-19b-3p target genes were analyzed using miRNA target prediction websites miRNA.org (www.microrna.org/microrna/home.do) and PicTar (http://pictar.mdc-berlin.de/) (Fig. 6a). We identified TNFAIP3 as a candidate target gene, which includes a seed sequence at the 3′UTR region that is complementary to a sequence within the 5′ end of miR-19b-3p. Moreover, we obtained the expression of miR-19b-3p and TNFAIP3 in 516 head and neck squamous carcinoma tissues from TCGA. Negative correlation between miR-19b-3p and TNFAIP3 was also observed (Additional file 3) (Fig. 6b). In addition, the TNFAIP3 mRNA regions complementary to these 6–8 nt seed sequences of miR-19b-3p were highly conserved among the different species (Fig. 6c).

To further determine whether miR-19b-3p regulates the translation of TNFAIP3, we transfected CNE1 and CNE2 cells with miR-19b-3p mimic and NC mimic, whereas NPC cells were transfected with miR-19b-3p inhibitor and NC inhibitor. qRT-PCR showed that the enhanced levels of miR-19b-3p significantly decreased expression of TNFAIP3 mRNA and that downregulated miR-19b-3p conversely increased expression of TNFAIP3 mRNA. Western blot analysis revealed that the protein level of TNFAIP3 was markedly reduced in the miR-19b-3p-expressing vector compared with the control vector (Fig. 6d). To further confirm whether the 3′UTR of TNFAIP3 mRNA is a potential functional target of miR-19b-3p, we generated full length TNFAIP3-3′UTR luciferase reporter vectors containing a predicted wild type binding site (TNFAIP3 wt 3′-UTR) or a mutated version (TNFAIP3 mut 3′-UTR). These vectors or control pCDNA3.1 vectors were co-transfected with either the miR-19b-3p or the miR-control vector, and luciferase activity was detected after 48 h. Overexpression of the miR-19b-3p reduced the luciferase activity of the TNFAIP3 wt 3′-UTR vector-transfected cells but not that of the TNFAIP3 mut 3′-UTR vector-or of the control pCDNA3.1 vector-transfected cells, whereas the control miR-vector could not significantly change the luciferase activity (Fig. 6e). These results reveal that miR-19b-3p could repress TNFAIP3 translation by targeting a specific 3′UTR mRNA binding sequence.

To clarify the mechanism of TNFAIP3 activity in radioresistance, biological functions of TNFAIP3 were subjected to KEGG Pathway analyses (Fig. 6f), which showed that TNFAIP3 is tightly associated with NF-κB signaling pathway, which plays an important role in the regulation of radiosensitivity.

### miR-19b-3p decreases radiosensitivity by targeting TNFAIP3

**Fig. 7:**
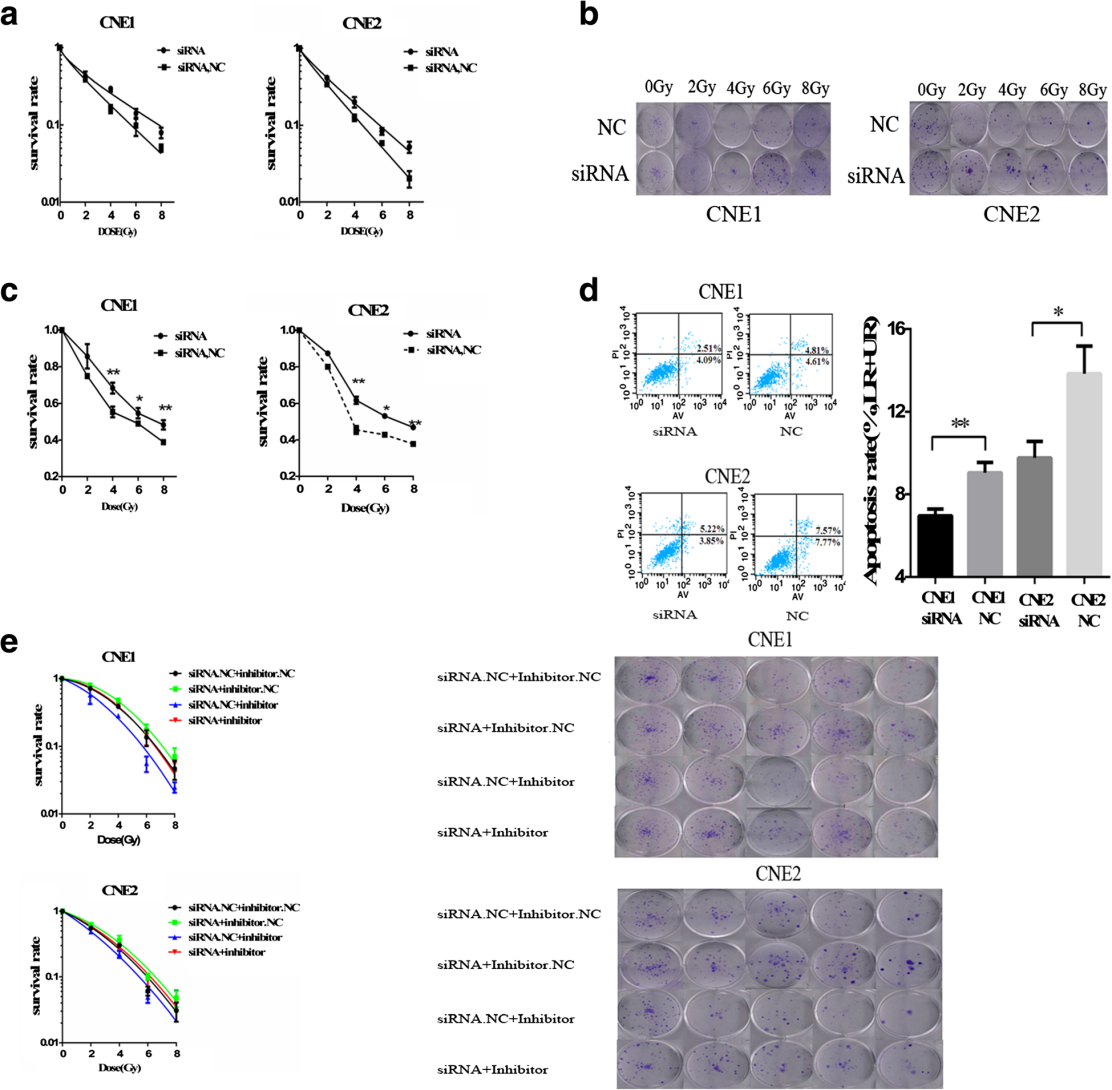
miR-19b-3p decreased radiosensitivity by targeting TNFAIP3. **a** Clonogenic survival assay showed that transfection of TNFAIP3 siRNA increased NPC cell radioresistance compared with control group after 2,4,6,8 Gy irradiation. **b** Representative image of colony formation in CNE-1 and CNE-2 cells exposed to different doses of IR. **c** Survival rates of NPC cells after 2,4,6,8 Gy irradiation were examined by CCK-8 assay. **d** Apoptotic changes in CNE-1 and CNE-2 cells transfected with si-TNFAIP3 after exposure to 4 Gy IR for 72 h. **e** Knockdown of TNFAIP3 restored the significant improvement in radiosensitivity in miR-19b-3p-inhibitor-transfected CNE1 and CNE2 cells after 2,4,6,8 Gy irradiation

To demonstrate the precise functions of TNFAIP3, we transiently transfected CNE-1 and CNE-2 cells with the siR-TNFAIP3 or siRNA control. A clone survival assay showed that knockout of TNFAIP3 markedly decreased radiosensitivity in the NPC cells [AUC 2.73 (CNE1.siRNA) vs. 2.35 (CNE1.NC), RPF = 1.17 AUC 2.43 (CNE2.siRNA) vs. 2.07 (CNE2.NC), RPF = 1.17; *p < 0.05*] (Fig. 7a and b). In addition, the survival rate of CNE1 and CNE2 cells with TNFAIP3 loss was increased after 2,4,6,8 Gy irradiation stimulation, as detected by CCK-8 assay (Fig. 7c). Knockout of TNFAIP3 in CNE1 and CNE2 cells also significantly decreased the percentage of apoptosis after irradiation (Fig. 7d). We then performed colony formation assays by co-transfecting miR-19b-3p inhibitor and siR-TNFAIP3 into NPC cell lines, which showed that decreased expression of TNFAIP3 restored the significant improvement in radiosensitivity by downregulating miR-19b-3p expression [AUC 3.585 (CNE1.inhibitor plus siRNA) vs 2.857 (CNE1.inhibitor plus siRNA.NC), RPF = 1.25 AUC 2.99 (CNE1.inhibitor plus siRNA) vs 2.54 (CNE1.inhibitor plus siRNA.NC), RPF = 1.18; *p < 0.05*] (Fig. 7e).

### miR-19b-3p increased the activity of NF-κB after irradiation stimulation

**Fig. 8:**
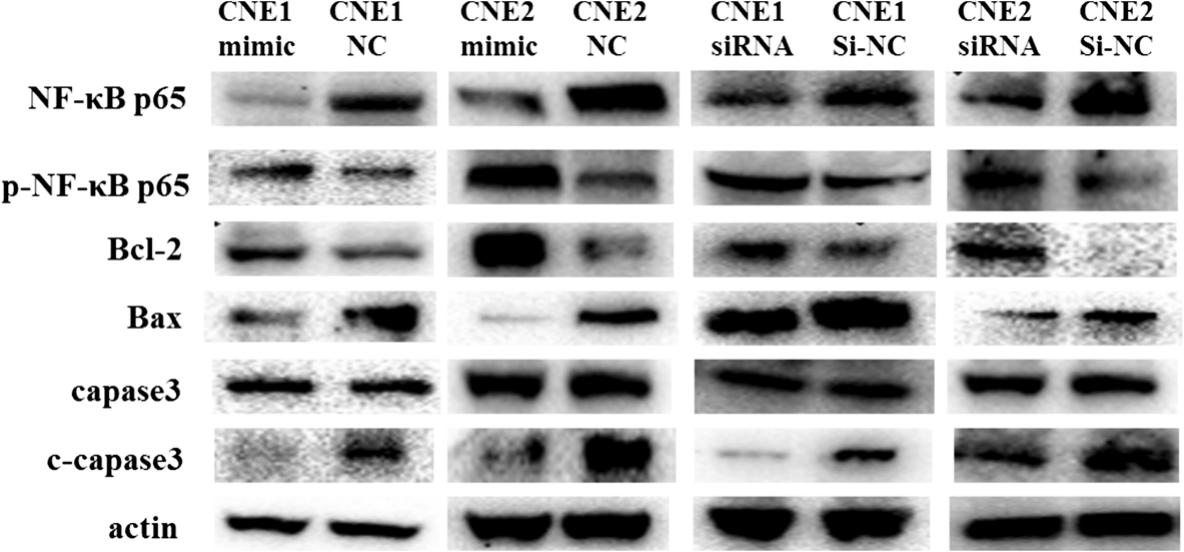
miR-19b-3p increased the activity of NF-κB by targeting TNFAIP3. CNE1 and CNE2 cells were transfected with miR-19b-3p mimic or siRNA against TNFAIP3 before irradiation stimulation. NF-κB, p-NF-κB, Bcl-2, Bax, caspase3, and c-capase3 were detected by Western blot

Having determined that miR-19b-3p was able to regulate NPC radioresistance, its effects on cell apoptosis were analyzed by transfecting CNE1 and CNE2 cells with miR-19b-3p mimic or TNFAIP3 siRNA, respectively, before radiation stimulation. Through bioinformatic analysis, both TNFAIP3 and miR-19b-3p were found to have a correlation with the NF-κB pathway. Previous studies indicate that activated NF-κB plays an important role in radioresistance [18]. Therefore, we investigated whether miR-19b-3p mediates NF-κB signaling pathway by targeting TNFAIP3 and progressively regulated NPC cell radioresponse. In either TNFAIP3 knockdown or transfection of miR-19b-3p mimic, we obtained similar results after 6 Gy radiation stimulation, as measured by Western blot (Fig. 8). Specifically, p65 level was decreased, and p-p65 level was increased in transfected cell lines compared with control groups. Because NF-κB is a known transcription factor of Bcl-2, we sought to examine the Bcl-2 expression level by Western blot, which revealed that Bcl-2 level was increased in the transfected cells. We also detected for Bax and c-capase3 levels, which both decreased in the transfected groups compared with the control groups. These studies show that miR-19b-3p can increase NF-κB activity by targeting TNFAIP3.

## Discussion

With the development of technology, intensity-modulated radiotherapy (IMRT) has improved locoregional control and overall survival of NPC compared with crude two-dimensional radiotherapy techniques and is now the preferred choice of therapy for NPC [1, 19]. However, NPC cannot be controlled in about 10% of patients receiving standard dose of radiation therapy; higher doses of radiation therapy produce severe toxicity [1]. Elucidation of the regulatory mechanism underlying radioresistance provides the basis for solving this issue. In the present study, we found that miR-19b-3p regulates the radioresistance of NPC through the NF-κB signaling pathway by targeting TNFAIP3 in vitro.

miRNAs, similar to genes, are classified as tumor-causing (called “oncomiRs”) or tumor-suppressive (“tumor-suppressive miRNAs”) based on their roles in cancer [2]. miR-19b has been shown to have a preponderant role in oncogenicity, and is an essential regulator related to tumor malignant biobehavior. miR-19b can enhance migration and invasion of lung cancer cells [20] and is associated with drug tolerance [21]. In cellular proliferation, however, miR-19b elicits different effects in different tissues; miR-19b accelerates melanoma cell proliferation, whereas it elicits the opposite effect in hepatic stellate cell [12, 22]. However, the function of miR-19b-3p in radioresistance is hardly studied. In the present study, we found that miR-19b-3p is upregulated in NPC and is associated with worse patient prognosis. Our data revealed that miR-19b-3p contributes to radioresistance in vitro and in vivo. miRNAs exert significant biological functions by regulating various target genes [23]. Then, via the miRNA target prediction program, TNFAIP3 is predicted as a potential target with the highest predictive value for miR-19b-3p. We further verified that miR-19b-3p regulates TNFAIP3 expression level by targeting the 3′-UTR of TNFAIP3 mRNA.

TNFAIP3 is widely considered as an important regulator of inflammation and immunity, and plays a key role in the modulation of immune response to pathogens as well as the development of inflammatory and autoimmune disease [24, 25]. Moreover, TNFAIP3 is now accepted as a disease susceptibility gene that may serve as a predictive biomarker [26]. However, the potential roles of TNFAIP3 in regulation of radiosensitivity are rarely studied. Our data demonstrated that knockout of TNFAIP3 can decrease NPC cell radiosensitivity. Furthermore, TNFAIP3 knockdown abolished radiosensitization induced by transfection of miR-19b-3p inhibitor in NPC cells. Overall, miR-19b-3p regulated nasopharyngeal carcinoma radioresistance by targeting TNFAIP3.

**Fig. 9:**
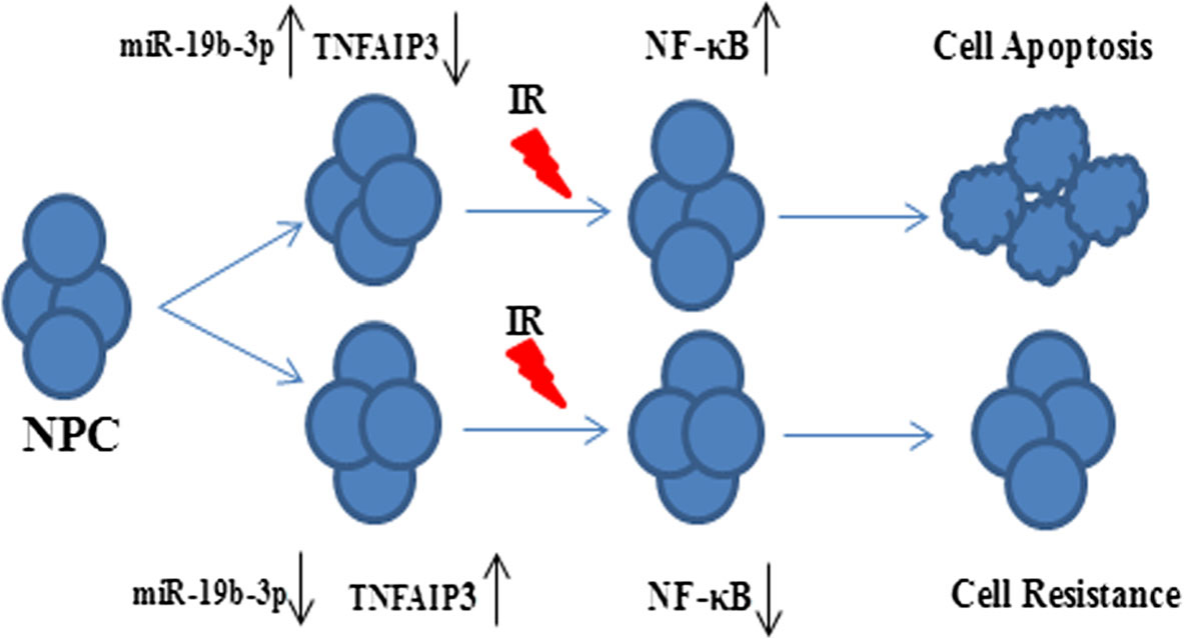
miR-19b-3p increased the radioresistance of NPC

Apoptosis is an important mechanism for the regulation of sensitivity to radiation [9, 27]. We found that overexpression of miR-19b-3p or knockout of TNFAIP3 decreased apoptosis rates of NPC cell lines after irradiation. Using bioinformatic analyses, we found that both miR-19b-3p and TNFAIP3 have a common apoptotic pathway—the NF-κB signaling pathway. Ionizing radiation is known to cause DNA DSBs and leads to lethal injury [9, 28]. IR-induced DSBs trigger a DNA damage response with NF-κB activation as the antiapoptotic factor in most cell types [29–32]. Thus, through the inhibition of NF-κB activation, radiosensitivity can be increased [33]. Previous studies have demonstrated that TNFAIP3 acts as a repressor that downregulates NF-κB activity. Indeed, we found that NF-κB activity was enhanced after radiation stimulation by the knockout of TNFAIP3. Furthermore, we confirmed that NF-κB activity improved after radiation stimulation by overexpressing miR-19b-3p. To further investigate the possibility of the mitochondrial signaling pathway as a molecular pathway for miR-19b-3p, the expressions of Bcl-2, Bax, capase3, and c-capase3 markers were examined. Our data revealed similar protein results from the NPC cell lines after either TNFAIP3 knockdown or transfection of miR-19b-3p mimic. miR-19b-3p increased the radioresistance of NPC by increasing NF-κB activity through TNFAIP3 targeting (Fig. 9).

## Conclusions

We revealed that miR-19b-3p is upregulated in NPC and serves as a regulator of NF-κB activity by targeting TNFAIP3. The newly identified miR-19b-3p/TNFAIP3/NF-κB axis sheds light on a novel molecular mechanism for NPC cell radioresistance, indicating that miR-19b-3p is a valuable biomarker and a promising therapeutic target for the management of NPC.

## Additional files

Additional file 1: Sample ID. (XLSX 11 kb)
Additional file 2: Correlation between the expression of miR-19b-3p and clinical features. (DOCX 18 kb)
Additional file 3: Sample ID. (XLSX 26 kb)

## Abbreviations

AUC: Area under the curve
CI: Confidence interval
DSBs: DNA double-strand breaks
HR: Hazard ratio
IR: Ionizing radiation
MID: Mean inactivation dose
miRNA: MicroRNA
NPC: Nasopharyngeal carcinoma
ROC: Receiver operating characteristic
RPF: Radiation protection factor
