# Supplementary material for: MicroRNA-19b-3p regulates nasopharyngeal carcinoma radiosensitivity by targeting TNFAIP3/NF-κB axis
Source: J Exp Clin Cancer Res. 2016 Dec 5;35:188. doi: 10.1186/s13046-016-0465-1 (PMC5139034; doi:10.1186/s13046-016-0465-1)
Supplement: Additional file 2: — Correlation between the expression of miR-19b-3p and clinical features. (DOCX 18 kb) [file 13046_2016_465_MOESM2_ESM.docx]

|  |  | miR-19b-3p expression level | |  |
| --- | --- | --- | --- | --- |
|  | All cases | Low expression | High expression | P value |
| Gender |  |  |  |  |
| Female | 33 | 10(30.3%) | 23(69.7%) | 0.873 |
| Male | 104 | 30(28.8) | 74(71.2%) |  |
| Age at diagnosis |  |  |  |  |
| ≤ 61 | 70 | 20(28.5%) | 50(71.5%) | 0.869 |
| >61 | 67 | 20(29.8%) | 47(70.2%) |  |
| T classificaction |  |  |  |  |
| T_1_-T_2_ | 52 | 13(25%) | 39(75%) | 0.398 |
| T_3_-T_4_ | 85 | 27(31.7%) | 58(68.3%) |  |
| N classificaction |  |  |  |  |
| N_0-1_ | 96 | 34(35.4%) | 62(64.6%) | 0.014 |
| N_2-3_ | 41 | 6(19.5%) | 35(80.5%) |  |
| Neoplasm histologic grade |  |  |  |  |
| G_1_-G_2_ | 99 | 31(31.3%) | 68(68.7%) | 0.426 |
| G_3_-G_4_ | 38 | 9(23.7%) | 28(76.3%) |  |

**Correlation between the expression of miR-19b-3p and clinical features**
